# Mannose Derivatives as Anti-Infective Agents

**DOI:** 10.3390/ijms262010230

**Published:** 2025-10-21

**Authors:** Rosana Ribić

**Affiliations:** Department of Nursing, Univeristy Center Varaždin, University North, Jurja Križanića 31b, 42000 Varaždin, Croatia; rribic@unin.hr; Tel.: +385-42-493-306

**Keywords:** mannose, glyconjugates, glycomimetics, anti-adhesion

## Abstract

Mannose is a natural monosaccharide that plays a central role in host–pathogen interactions and has emerged as a versatile scaffold for designing anti-infective agents. This review summarizes recent advances in mannose-based glycoconjugates with antibacterial, antiviral, antifungal, and antiparasitic activity. In bacteria, FimH antagonists prevent *Escherichia coli* adhesion, while mannose-functionalized materials disrupt *Pseudomonas* and *Burkholderia* biofilms or enhance delivery of anti-tubercular drugs. In virology, mannose-containing dendrimers, glycopolymers, and nanoparticles inhibit HIV, SARS-CoV-2, Ebola, HPV, and HSV by targeting viral glycoproteins or blocking lectin-mediated transmission. Mannose-decorated vaccines and nanocarriers also show promise against fungal pathogens and parasites. Continued optimization of presented structures could lead to the promising candidates for clinically applicable therapies.

## 1. Introduction

Carbohydrates serve essential roles such as modifying biomolecules for specific functions, mediating metabolic processes, acting as biosynthetic intermediates, and most notably, supporting energy generation and storage. Their covalent linkage to proteins provides a structural scaffold necessary for proper protein function. Carbohydrate-based modifications are crucial for processes such as cell–cell communication and adhesion, protein conformation, membrane architecture, and intracellular signaling [1].

**Figure 1:**
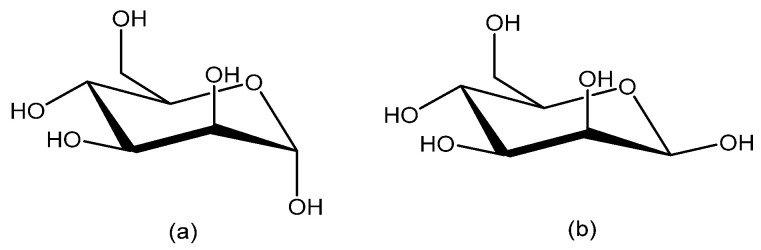
d-Mannose in cyclic forms; (**a**) α-mannopyranose and (**b**) β-mannopyranose.

Carbohydrate-binding agents (CBAs) exploit these glycan structures to modulate or interfere with biological interactions, making them valuable tools in both therapeutic and diagnostic applications. CBAs refer to natural or synthetic molecules that bind specifically to carbohydrates (sugars) or glycan structures on biomolecules, enabling them to block host–pathogen interactions, label cells, or activate immune responses. Main classes of CBA are lectins, antibodies and synthetic CBAs like glycomimetics [2,3].

d-Mannose is a naturally occurring hexose sugar (Figure 1), primarily found in plant cell walls as a component of mannan. It plays a critical role in glycoprotein synthesis, contributing to immune regulation, wound healing, antimicrobial and antitumor activities [4]. Recent studies highlight mannose’s potential in immunoregulation, suppressing autoimmune and inflammatory conditions. Additionally, mannose-based delivery systems have been developed for targeted therapies [5].

Since mannose is a key sugar involved in microbial recognition in humans, this review focuses on glycoconjugates containing mannose (mannoconjugates) that act on biological targets exhibiting antiviral, antibacterial, antifungal, and antiparasitic activities. The goal is to consolidate the most significant recent findings related to mannoconjugates as anti-infective agents, highlighting their emerging potential as drug candidates. Emphasis is placed on mannose-targeted pathways, which are receiving growing attention due to their biological and clinical relevance. Glycodendrimers are a notable category of synthetic macromolecules designed to replicate various structural and functional aspects of cell-surface glycoconjugates. The carbohydrate components of these molecules play crucial roles in the processes of bacterial and viral infections, primarily through carbohydrate–protein interactions [6]. Research has demonstrated that the molecular architecture, valency, and spatial arrangement of carbohydrate epitopes significantly influence the specificity and strength of these interactions. Selecting appropriate glycodendrimers can effectively disrupt these interactions, thereby preventing bacterial or viral adhesion and entry into host cells—an approach that offers a promising means of infection control. This review highlights recent advances in the design of glycodendrimers containing mannose developed for anti-infective therapies [7].

## 2. Antibacterial Activities

### 2.1. Escherichia coli

Recurrent urinary tract infections (UTIs) represent a significant clinical challenge, particularly among adult women, due to their high prevalence and the limitations associated with antibiotic prophylaxis, including antimicrobial resistance and adverse effects [8]. The widespread reliance on antibiotics as first-line therapy further exacerbates the problem by driving the emergence of antimicrobial resistance, with *Escherichia coli* constituting a major contributor to the global antimicrobial resistance burden [9]. Consequently, there is a growing demand for novel strategies to prevent and treat UTIs, among which anti-adhesion therapy has emerged as one of the most promising approaches [10,11].

Recent systematic reviews and meta-analyses have evaluated the efficacy of d-mannose, as a non-antibiotic option for preventing and managing UTIs, particularly those caused by uropathogenic *E. coli* (UPEC). d-mannose significantly lowers the risk of recurrent UTI compared to placebo and shows similar efficacy to commonly used antibiotics like nitrofurantoin [12]. Importantly, d-mannose exhibits a favorable safety profile, with less adverse events reported in comparison to antibiotics. These findings position d-mannose as an effective and well-tolerated prophylactic agent in UTI management, contributing to reduced antibiotic consumption and helping combat the global challenge of antimicrobial resistance [13].

The pathogenesis of UTIs caused by *E. coli* critically depends on the bacterium’s ability to adhere to the epithelial cells lining the urinary tract. Namely, the initial step in UTI pathogenesis involves the binding of bacterial surface-associated proteins, known as adhesins, to complementary receptors on eukaryotic cells. These adhesins are often presented on filamentous surface structures termed pili or fimbriae. In UPEC, the most prevalent adhesive organelles include type 1, P, S, and F1C fimbriae [10]. Type 1 pili are complex protein assemblies consisting of a linear tip fibrillum at its distal end, composed of the mannose-binding adhesin FimH together with the adaptor subunits FimG and FimF. This fibrillum is anchored to a helical rod made up of several hundred to several thousand FimA subunits, with assembly terminated by a single FimI subunit at the proximal end and capped by the chaperone FimC. The outer membrane usher protein FimD serves as the assembly platform and orchestrates pilus biogenesis [14].

FimH is composed of the N-terminal lectin-binding domain (FimH_LD_) encompassing the carbohydrate-binding domain (CBD) which recognizes mannose residues on host cell glycoproteins, and C-terminal pilin domain (FimH_PD_). FimH_LD_ goes through the conformational changes with two endpoint conformations; an “open” conformation that binds mannose weakly and a “closed” conformation that binds with a high affinity [15]. The pilin domain fine-tunes this switch, usually holding the lectin domain in the low-affinity state under static conditions. When mannose derivatives are encountered, however, the lectin domain shifts into its tightly binding form, increasing its affinity up to 100,000-fold [16]. Natural ligand for FimH is a heavily mannosylated oligosaccharide (Man_9_GlcNAc_2_). Because this molecular system is central to bacterial adhesion and colonization, FimH has become a prime focus for developing anti-adhesives and vaccines aimed at preventing UTIs [17]. FimH is also crucial factor in the pathogenicity of adherent-invasive *E. coli* (AIEC). By binding to mannosylated receptors such as CEACAM6 on the intestinal epithelium, FimH enables AIEC to strongly adhere to and colonize the gut mucosa. This interaction promotes bacterial invasion, persistence, and the induction of inflammatory responses characteristic of Crohn’s disease. Because of its central role in AIEC adhesion, FimH is considered a promising therapeutic target for strategies aimed at preventing or treating AIEC-driven intestinal inflammation [18].

Research into FimH antagonists began decades ago. In 1977, Ofek, Mirelman, and Sharon provided a detailed description of mannose’s inhibitory effects on *E. coli* [19]. Since then, significant progress has been made in designing both mono-glycomimetics and multivalent glycomimetic constructs that exhibit enhanced binding affinity and specificity for FimH. α-d-Mannopyranosides with hydrophobic aglycons are potent FimH inhibitors [10,20,21]. Simple α-d-mannopyranoside, *n*-heptyl mannoside **1**, exhibits strong binding with a K_D_ of 5 nM [22], while simple aryl mannosides displayed weaker binding, among them *ortho*- and *meta*-substituted derivatives showed higher potency than their corresponding *para* analogues.

**Figure 2:**
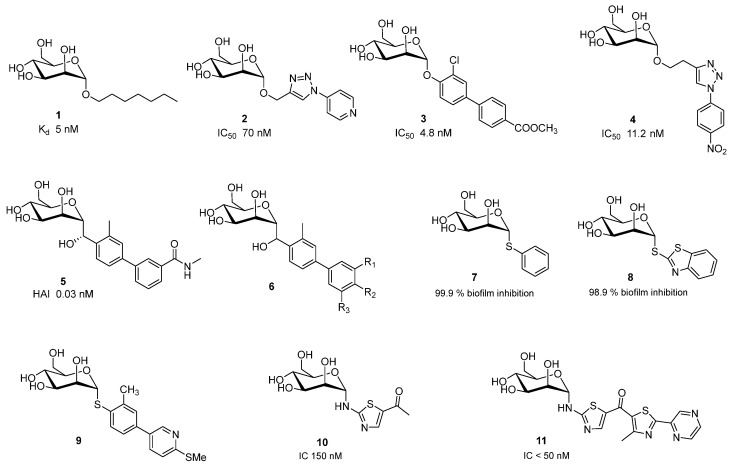
Potent monovalent FimH antagonist.

Namely, based on co-crystal structures of *E. coli* FimH in complex with mannopyranoside antagonists, researchers identified mannopyranoside derivatives with hydrophobic aglycons as promising candidates due to their strong binding affinity [23,24,25]. This conclusion was drawn from the hydrophobic interactions observed within CRD of FimH. In particular, interactions involving Tyr-48, Tyr-137, and Ile-52 residues from the “tyrosine gate” within the active binding site [25]. Therefore, addition of a second aryl ring increases hydrophobic and π-stacking interactions within the tyrosine gate, as shown in representing *O*-mannosides **2**,**4** [26], **3** [27] (Figure 2). Researchers have also explored *C*-, *N*-, and *S*-mannosides because a common limitation of *O*-mannosides is their poor bioavailability due to the instability of the *O*-glycosidic bond. *C*-mannosides **5** [28], and GSK3882347 **6** showed great potential and it should be highlighted that GSK3882347 **6** was in clinical trial for UTI [29].

Abdu-Allah et al. also evaluated thiomannosides and showed that their activity was primarily influenced by the nature of the aryl or heteroaryl aglycone, and that compounds **7** and **8** (Figure 2) had superior affinity and inhibitory effects then the reference ligand **1** [30].

Nishi et al. have prepared and evaluated thiomannoside **9** with improved metabolic stability and plasma exposures, and potential to inhibit biofilm formation and also to disrupt the preformed biofilm [31].

Gouin et al. showed that thiazolylaminomannosides **10** and **11** (Figure 2) have nanomolar affinity for FimH. Especially, compound **11** which have high capacity of to prevent AIEC adhesion in vitro and ex vivo compared to **1** and α-d-mannose [26]. Second generation of homologated *C*-mannosides with an NH group exhibited anti-adhesive effect lower than the first class of *N*-linked mannosides [32].

**Figure 3:**
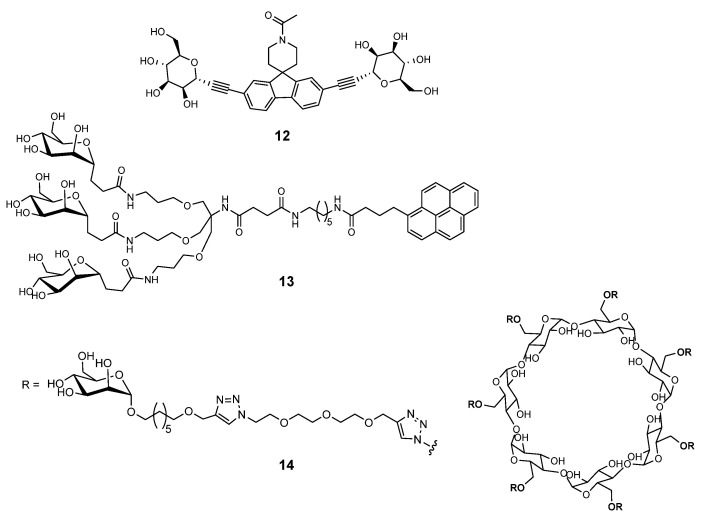
Multivalent FimH antagonists.

Structures harboring di-, tri-, hepta- and dendrimetic α-mannopyranosides **12**–**14** shown in Figure 3 are also very potent FimH antagonists [21], while the divalent C-linked mannopyranoside EB8018/Sibofimloc **12** which entered Phase IIa clinical trials against Crohn’s disease [33].

In general, small-molecule inhibitors have been optimized to improve oral bioavailability, metabolic stability, and target selectivity, showing promising anti-adhesive properties in vitro and in vivo models. Multivalent glycomimetics, such as dendrimers and nanoparticles functionalized with mannose residues, demonstrate multivalent interactions with bacterial lectins, greatly increasing their inhibitory potency against *E. coli* adhesion and biofilm formation [34,35]. These constructs not only improve bacterial clearance but also reduce the risk of resistance development by blocking bacterial colonization rather than killing the bacteria directly. Continued efforts in refining molecular design and delivery methods are essential to advance these agents toward clinical application, offering alternatives to conventional antibiotics and addressing rising antimicrobial resistance [21].

### 2.2. Pseudomonas aeruginosa and Burkholderia cepacia Complex

Chronic lung colonization by opportunistic pathogens such as *P. aeruginosa* and members of the *B. cepacia* complex (Bcc) remains a leading contributor to illness and death in cystic fibrosis patients.

#### 2.2.1. *Pseudomonas aeruginosa*

Infections caused by *P. aeruginosa*, particularly in hospitalized and cystic fibrosis patients, are notably difficult to treat due to the bacterium’s ability to form antibiotic-resistant biofilms. *P. aeruginosa* employs various adhesins, including LecA (PA-IL) and LecB (PA-IIL) lectins, type VI pili, and flagella, as well as iron acquisition systems to establish infection and form protective biofilms. The lectin LecB has been identified as a key factor in biofilm formation, and its inhibition via specific carbohydrate ligands has been shown to reduce biofilm accumulation. LecB is a tetrameric, soluble C-type lectin with affinity for l-fucose, d-mannose, and glycans presenting terminal fucosyl or mannosyl residues. The crystal structure of LecB in complex with fucose, mannose and fructopyranose was determined [36]. A defining characteristic of the carbohydrate-binding site is the presence of two Ca^2+^ ions. They stabilize the conformation of loop Asn95-Asp104, which forms the core of the carbohydrate-binding site. Upon binding of a monosaccharide, these ions form direct interactions with three hydroxyl groups of the sugar [36]. To unravel the molecular basis of this selectivity, a series of glycomimetic derivatives were designed. Structural and thermodynamic analyses revealed that perturbation of the hydrogen bonding network in mannosides contributes to their lower binding affinity [37].

**Figure 4:**
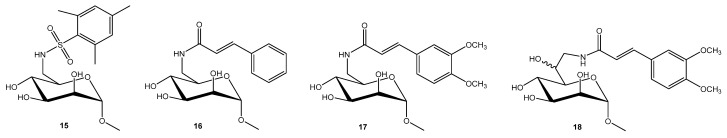
Potent monovalent LecB inhibitors.

Extensive research efforts have been directed on developing glycoconjugate-based inhibitors that target LecB lectin [38]. Titz et al. identified d-mannose derivatives bearing sulfonamide **15** and amide **16** substituents at C6 (Figure 4) as potent LecB inhibitors, effectively blocking bacterial adhesion with up to a 20-fold increase in affinity to LecB compared to the natural ligand methyl mannoside. Amide **16** is weaker LecB binder (IC_50_ 37 μM) then compound **15** (3.4 μM) because of dual binding contributions of sulfonamide group; (i) coordination of two to LecB Ca^2+^ ions with O4 and O5, and (ii) H-bond formed between the sulfonamide and Asp96, in addition to the lipophilic interaction of the aryl group with the binding pocket surface [39]. To optimize cinnamide inhibitors, over 20 derivatives were synthesized with varied aromatic substitutions. SAR analysis revealed that *ortho* substitutions reduced potency, likely due to steric clash, while *meta* and *para* lipophilic groups significantly improved activity. Polar substituents consistently weakened binding, confirming a strong preference for hydrophobic interactions within the LecB pocket. Methoxy emerged as the optimal substituent, and the dimethoxy derivative **17** was the most potent compound (IC_50_ = 19.9 µM). [40]

Furthermore, mannoheptose derivatives bearing cinnamide and sulfonamide groups were prepared by elongating the sugar backbone, with the aim of liberating the O6 position for potential hydrogen bonding with Ser23. However, compounds such as **18** did not exhibited improved affinity, even though (6*S*)-mannoheptose derivatives demonstrated higher inhibitory activity than methyl α-d-mannoside [41].

FThe fucosylated mannose-centered glycocluster also demonstrated good binding activity (in the low micromolar range). Antenna-like scaffolds were preferred over linear or crown-like architectures, and within crown-shaped carbohydrate-centered fucosylated glycoclusters, mannose-based cores outperformed those built on glucose or galactose [42,43].

Biofilms present a major barrier to effective treatment of bacterial infections, shielding pathogens from both host immune responses and antibiotic therapies. This protective matrix enables chronic colonization and contributes significantly to antimicrobial resistance. One strategy for eliminating biofilm involves glycan-targeted polymers that selectively bind biofilm structures. Using a continuous-flow biofilm model with *P. aeruginosa* PAO1 strain, mannose-functionalized fluorescent polymers exhibited stable and prolonged association with biofilms for over 24 h. Genetic knockout studies further revealed that both the lectin LecB and the adhesin CdrA contribute to polymer retention, highlighting their role in the polymer–biofilm interaction [44].

**Figure 5:**
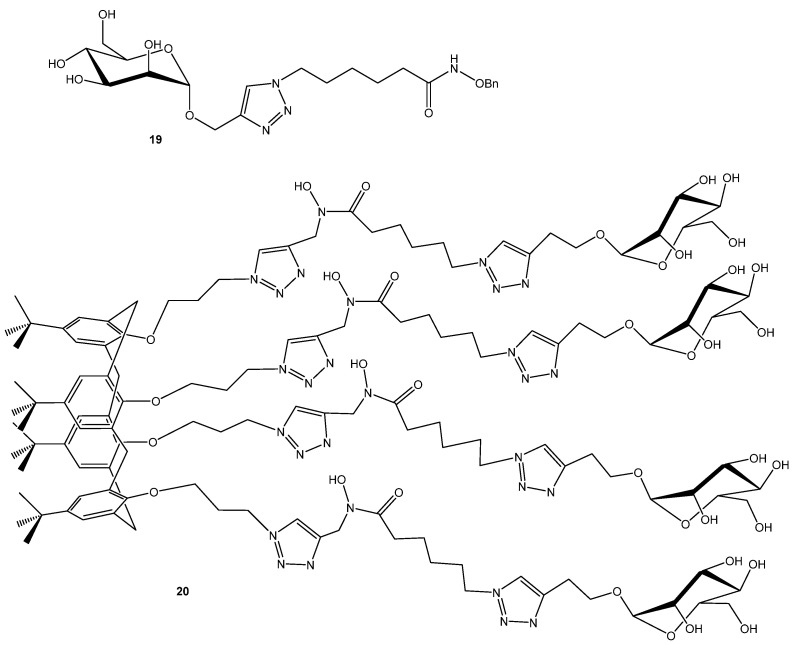
Multivalent LecB inhibitor.

Another approach is targeting iron uptake while simultaneously interfering with adhesin functions. O’Toole and Mo Benazza with coworkers designed an glycocluster tetrahydroxamic acid iron-chelating calixarene (Figure 5) that disrupts both iron sequestration and glycan-mediated adhesion [45]. In the fluorescence polarization competition assays monovalent mannose ligands (methyl α-d-mannopyranoside (IC_50_ 157 μM) and compound **19** (146 μM) displayed weak affinity relative to fucose analogues, while the mannose-capped multivalent cluster **20** exhibited a dramatic enhancement in binding (IC_50_ 0.66 μM). In contrast, the fucose-based cluster showed diminished potency upon multimerization. Surprisingly, the mannocluster outperformed the fucocluster, despite L-fucose being LecB’s natural ligand. The data suggest a chelating binding mode as the likely origin of this unexpected mannose-driven affinity enhancement [45].

#### 2.2.2. *Burkholderia cepacia* Complex

**Figure 6:**
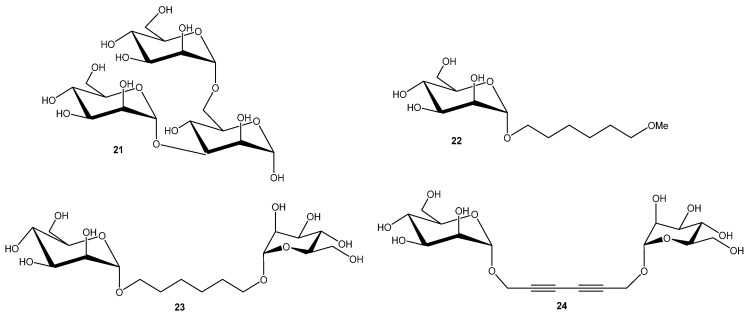
BC2L-A ligands.

*Burkholderia cenocepacia* is one of the most dangerous in the *B. cepacian* complex (Bcc). It is a Gram-negative, rod-shaped bacterium commonly found in soil and water but is also an opportunistic pathogen. In cystic fibrosis patients, it can cause chronic lung infections, and in severe cases, a fatal condition known as “cepacia syndrome”. The bacterium is highly resistant to antibiotics and capable of forming biofilms, making infections difficult to treat. *B. cenocepacia* produces three soluble lectins; BC2L-A, BC2L-B, and BC2L-C. Each have sequence similarity with PA-IIL. The smallest member of this family, BC2L-A, exhibits mannose specificity [46,47]. Each monomer contains a single CRD that coordinates two Ca^2+^ ions directly involved in ligand binding. Crystallographic analyses with methyl-α-d-mannopyranoside (αMeMan) [46] and a branched trimannoside (αMan1-3(αMan1-6)Man) [47] revealed that the high affinity results from the coordination of three mannose hydroxyl groups (O-2, O-3, and O-4) by the two Ca^2+^ ions in the binding site. Trisaccharide (αMan1-3(αMan1-6)Man **21** (Figure 6) was tested by titration microcalorimetry in order to evaluate their affinity for BC2L-A in solution, along with disaccharides (αMan1-2Man, αMan1-3Man, αMan1-6Man), and synthetic mono- and dimannnosides. Natural dimannosides showed only modest variation in LecB affinity (αMan1-3Man was the strongest (Kd = 2.6 μM)). In contrast, synthetic mono-mannoside **22** and dimannoside with flexible linker **23** behaved similarly to αMan1-3Man, with moderate affinity (Kd = 2.8 μM). The rigid analogue **24** exhibited substantially enhanced affinity (Kd = 220 nM) and a stoichiometry of ~0.5, indicating simultaneous engagement of both mannose units via a bridging mode. Thus, ligand rigidity strongly promotes multivalent binding and affinity enhancement [47].

Marchetti with coworkers have demonstrated that revealed that a manno configuration bearing a hydroxyl or glycol group at C6 is essential for BC2L-A recognition [48].

**Figure 7:**
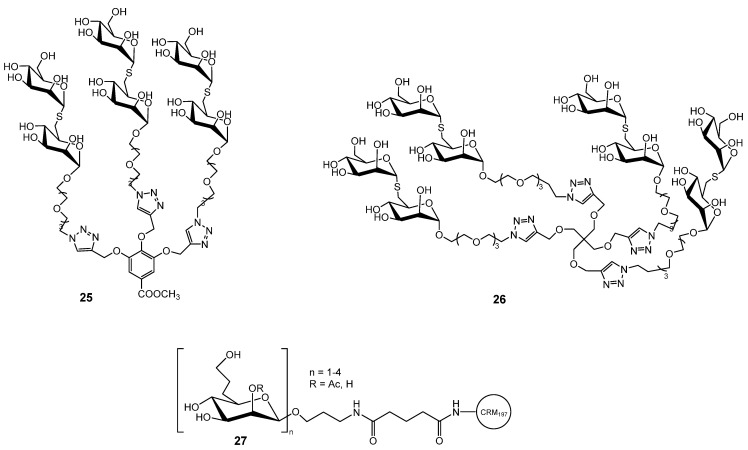
Multivalent mannose derivatives explored against *B. cenocepacia*.

To exploit the cluster glycoside effect, multivalent mannoside derivatives were also developed. Since replacing the glycosidic oxygen with sulfur increases resistance to chemical and enzymatic hydrolysis, Wimmerová, Borbás and co-workers synthesized multivalent thiomannosides (Figure 7) with an (α1→2)-thio-linked mannobioside mimic functionalized with an azide-bearing aglycone, which was subsequently conjugated to various multivalent scaffolds via copper-catalyzed azide–alkyne cycloaddition. The methyl gallate-based cluster **25** and pentaerythritol-based glycoclusters **26** showed only slightly higher inhibition potency than αMeMan (relative potency 1.3), whereas compound 14 exhibited more than double the activity [49].

Multivalent mannose derivatives were also evaluated for their potential application in anti-*B. pseudomallei* and anti-*B. mallei* vaccines. 6-Deoxy-d-manno-heptopyranose and its *β*-(1→3)-linked oligomers **27** (Figure 7), polysaccharides common to *B. pseudomallei* and *B. mallei*, were prepared. Synthetically, the most challenging task was the preparation of β-mannosidic linkages within the oligosaccharides, which was achieved with high stereoselectivity through hydrogen-bond-mediated aglycone delivery. They were used for subsequent conjugation to the carrier protein CRM197, enabling immunological evaluation. These glycoconjugates elicited strong IgG antibody titers and robust T cell–dependent immune responses in mice [50]. Another example of mannose targeted delivery system is amphiphilic diblock copolymer, poly(ethylene oxide)-*b*-poly(ε-caprolactone) (PEO-*b*-PCL), functionalized with a terminal mannose group. The mannose moiety was subsequently introduced via quaternization of the PEO chain end with a brominated mannose derivative. These amphiphilic copolymers self-assembled into uniform spherical micelles with the mannose units on the micelle surface that served for the binding of BC2L-A lectin. The binding thermodynamics confirmed selective interaction, highlighting the potential of these micellar systems in targeted drug delivery and vaccine applications [51].

### 2.3. Mycobacterium tuberculosis

The global health threat posed by tuberculosis (TB) has been intensified by the emergence of multidrug-resistant strains of *M. tuberculosis* (Mtb) and a long-standing stagnation in the discovery of novel mycobactericidal agents. A key factor underlying the persistence and resilience of Mtb is its unusually impermeable and polysaccharide-rich cell wall, a complex structure that plays a critical role in virulence and drug resistance. Despite its importance, many aspects of mycobacterial cell wall biosynthesis remain incompletely understood. Among the enzymes involved in cell wall construction is glucosyl-3-phosphoglycerate synthase (GpgS), a retaining glycosyltransferase found in Mtb and related species. GpgS is unique in that it belongs to the newly defined GT-81 family and shares low sequence similarity with previously characterized glycosyltransferases. Functional studies have confirmed its essentiality for Mtb survival, highlighting it as a potential drug target. The three-dimensional representations of GpgS, including the apo form and its ternary complex with UDP and 3-phosphoglycerate, was resolved by X-ray crystallography [52]. Importantly, the structural data illuminate the enzyme’s specificity for UDP-based donor substrates and its preference for glucose over mannose, while identifying key residues responsible for acceptor substrate recognition [53,54].

A severe extrapulmonary manifestation of Mtb infection, presents unique therapeutic challenges. Current treatment protocols rely on prolonged administration (8–20 months) of first- and second-line anti-tubercular drugs, typically delivered orally or intravenously at high doses due to limited bioavailability. The long-term multidrug regimen required for TB treatment is often accompanied by the emergence of drug-resistant Mtb strains [55]. Nanocarrier-based drug delivery systems offer notable advantages, including improved bioavailability, reduced systemic toxicity, and lower dosing frequency, which collectively contribute to better patient compliance [56]. However, passive targeting through nanoformulations alone often lacks sufficient specificity. To address this limitation, active targeting strategies have emerged, where nanocarriers are conjugated with ligands that bind selectively to receptors or cellular components of macrophages, the primary host cells for Mtb, or to structures unique to the mycobacterial cell wall [57]. Ligand-mediated targeting not only improves drug accumulation at intracellular sites of infection but can also enhance adhesion to mucosal surfaces such as alveolar or intestinal epithelium, especially through interactions with lectin-binding domains. A wide array of ligands has been investigated for this purpose, including mannose, mycolic acid, lectins, and aptamers, each offering distinct advantages in directing drug-loaded nanocarriers to desired biological targets [58].

Polymeric nanoparticles, particularly those composed of biodegradable materials like chitosan, are readily internalized by macrophages through opsonization and phagocytosis. Targeting can be further enhanced by surface modification with ligands such as mannose, which exploits receptor-mediated uptake mechanisms specific to macrophages. In recent work, an intra-articular injectable, in situ gelling system incorporating mannose-conjugated chitosan nanoparticles was developed for localized and sustained delivery of anti-tubercular drug rifampicin [59].

Another promising strategy for targeted delivery of rifampicin involves the use of mannose-anchored solid lipid nanoparticles (SLNs). Antimicrobial assays revealed a notable enhancement in activity, with MICs reduced 4-fold and 8-fold against wild-type and drug-resistant *Mycobacterium smegmatis*, respectively, compared to free rifampicin. Fluorescently labeled SLNs (loaded with coumarin-6) further demonstrated improved macrophage uptake following mannose conjugation, confirming receptor-mediated internalization. Importantly, mannose-rifampicin-SLNs exhibited superior intracellular killing of *M. tuberculosis* H37Ra within macrophages, outperforming both non-functionalized SLNs and free rifampicin [60].

### 2.4. Methicillin-Resistant Staphylococcus aureus (MRSA)

Methicillin-resistant *S. aureus* (MRSA) is a leading cause of osteomyelitis. The clinical management of hospital-acquired chronic osteomyelitis caused by MRSA is hindered by two major obstacles: poor drug penetration into deep tissues and the rapid onset of an immunosuppressive microenvironment. MRSA has the ability to evade immune detection and impair both innate and adaptive immune responses [61]. To enhance antibiotic efficacy and eliminate intracellular bacteria, researchers developed different antibiotic delivery system targeting macrophages. The mannose-modified nanotherapeutic system co-delivering Zn^2+^ and vancomycin (Van) demonstrated potent antibacterial activity against both extracellular and intracellular MRSA, significantly lowering the minimum inhibitory concentration (MIC) of vancomycin. In vivo studies using a murine osteomyelitis model confirmed that Man-Zn^2+^/Van nanoparticles effectively reduced bacterial burden, restored normal gait, enhanced bone regeneration, and suppressed pro-inflammatory cytokine levels. Mechanistically, the nanotherapeutic acts through multiple antibacterial pathways, including disruption of the MRSA cell membrane, degradation of intracellular proteins and DNA, suppression of glycolysis, and interference with bacterial energy metabolism [62].

Another example is nanovesicle-based system that integrates macrophage-derived microvesicles with an M1 pro-inflammatory phenotype (M1-MW), which encapsulate vancomycin-crosslinked micelles containing the sonosensitizer indocyanine green (termed VCG micelles). The surface of M1-MW was modified with PEGylated mannose, enabling targeted delivery to infected tissues. In the reductive microenvironment typical of infections, these micelles release indocyanine green, which produces reactive oxygen species (ROS) upon ultrasound exposure, enhancing bacterial killing in conjunction with vancomycin. In an osteomyelitis mouse model, treatment with this platform led to improved survival, effective bacterial clearance, and reprogramming of macrophages toward the M1 phenotype [63].

Wang and He with coworkers described exosome-based antibiotic delivery system composed of mannosylated exosomes which are preferentially internalized by macrophages, loaded with lysostaphin (MExoL) and vancomycin (MExoV) [64]. The combined use of MExoL and MExoV effectively eradicated dormant intracellular MRSA. Furthermore, following intravenous administration, mannosylated exosomes rapidly accumulated in the liver and spleen, primary sites of intracellular MRSA infection, highlighting their organ-targeting capability [64].

### 2.5. Other Bacteria

Studies also highlight the potential of mannose-rich compounds in modulating bacterial adhesion and biofilm formation of *Clostridium difficile* and *Salmonella* [65,66]. In the *C. difficile* study, mannose, along with fructooligosaccharides, was shown to significantly inhibit bacterial adhesion to human epithelial cells, particularly across different strains. However, at sub-inhibitory concentrations, both fructooligosaccharides and mannose unexpectedly promoted biofilm formation, illustrating the complex, context-dependent effects of prebiotics on bacterial behavior [66]. In contrast, the mannose-rich oligosaccharides prevented bacterial adhesion of *Salmonella* to intestinal epithelial cells by binding to mannose-specific lectins on *Salmonella*. Moreover, mannose-rich oligosaccharides exhibited immunomodulatory effects, reducing inflammation and regulating energy metabolism in a chicken model of systemic inflammation induced by *Salmonella* [65]. Although these studies did not identify the exact lectin targeted by mannose structures, they emphasize the potential of mannose-containing compounds as non-antibiotic strategies for mitigating bacterial infections by targeting bacterial adhesion.

Due to the stagnation in antibiotic discovery and the rise of multidrug resistance, *Helicobacter pylori*-associated gastric infections have become increasingly difficult to treat. As an alternative therapeutic approach, chitosan was functionalized with mannose via reductive amination, followed by ionic gelation to produce mannose-functionalized chitosan nanoparticles (Man-CS-Nps). Molecular docking and molecular dynamics (MD) simulations were performed targeting *H. pylori* lectin, a protein implicated in bacterial adhesion, biofilm formation, and cytotoxicity. The antibacterial potential of Man-CS-Nps was evaluated through time-kill assays, polystyrene adherence tests, and antibiofilm studies, demonstrating significant anti-adhesion and biofilm-disrupting effects against resistant *H. pylori* strains [67].

### 2.6. Mannose-Containing Antibiotics

In clinical use for serious Gram-positive infection are natural glycopeptide teicoplanin and semi-synthetic lipoglycopeptides dalbavancin and oritavancin. Teicoplanin from *Actinoplanes teichomyceticus*, targets cell wall synthesis (d-Ala-d-Ala binding). It is effective in the treatment of Gram-positive infections (skin, bone, endocarditis), as an alternative to vancomycin [68]. Dalbavancin is derived from teicoplanin with dual mechanisms of action: It blocks bacterial cell wall synthesis and anchors to cell membranes. Compared to earlier glycopeptide, it shows enhanced activity against Gram-positive bacteria and has an extended half-life of about one week, persisting even longer in tissues such as skin and bone than in plasma [69]. Oritavancin also related to teicoplanin and dalbavancin, is approved for acute bacterial skin and skin structure infections [70].

Mannose-containing antibiotics which were studied in preclinical/experimental settings are actaplanin, ramoplanin, and mannopeptimycins. Actaplanin (A4696) is a broad-spectrum Gram-positive antibiotic complex produced by *Actinoplanes missouriensis*. It consists of several related actaplanins (A, B1, B2, B3, C1, G), all sharing the same peptide core and an amino sugar, but differing in the amounts of glucose, mannose, and rhamnose attached [71]. Ramoplanin is a lipoglycopeptide antibiotic complex derived also from *Actinoplanes* sp. It consists of three related polypeptides featuring chlorinated phenyl groups and d-mannose [72]. Mannopeptimycins are cyclic glycopeptide antibiotics isolated from *Streptomyces hygroscopicus*, consisting of a heptapeptide core decorated with mannose. Their mode of action includes binding lipid II, a key precursor in bacterial cell wall biosynthesis, thereby blocking peptidoglycan formation [73].

## 3. Antiviral Activities

Dendritic cells (DCs) are specialized antigen-presenting cells that detect and capture invading microbes at the skin or mucosal surfaces. They process these antigens into peptide fragments that are loaded onto major histocompatibility complex (MHC) molecules. Following antigen uptake, immature DCs gain the ability to migrate to lymph nodes, where they present the processed antigens to T cells, thereby initiating adaptive immune responses [74]. DCs are equipped with a diverse array of pathogen-recognition receptors (PRRs), such as Toll-like receptors (TLRs) and C-type lectins. These receptors play roles in sensing both self and foreign antigens, with some directly involved in pathogen recognition. C-type lectins, a broad family of Ca^2+^-dependent carbohydrate-binding proteins, specifically recognize microbial glycans. Among the many C-type lectins expressed by DCs, DC-specific intercellular adhesion molecule-3-grabbing non-integrin (DC-SIGN) is one well-characterized example. DC-SIGN is a type II transmembrane C-type lectin acting as a pattern recognition receptor, serving as a versatile entry receptor for numerous pathogens, including epidemic and pandemic viruses like Severe Acute Respiratory Syndrome Coronavirus 2 (SARS-CoV-2), Human Immunodeficiency Virus type 1 (HIV-1), and Ebola, Dengue and Zika viruses [75]. DC-SIGN binds mannose-containing structures in a Ca^2+^-dependent manner, promoting endocytosis and influencing the course of viral infection and immune activation [76].

### 3.1. Human Immunodeficiency Virus (HIV)

Acquired immunodeficiency syndrome (AIDS), caused by the human immunodeficiency virus (HIV), remains a major global health concern. According to Joint United Nations Programme on HIV/AIDS (UNAIDS), in 2024, about 40.8 million people were living with HIV, 1.3 million were newly infected, and 630,000 died from AIDS-related complications [77]. Current antiviral therapies primarily target viral enzymes—reverse transcriptase, protease, and integrase, as well as viral entry mechanisms [78]. Preventing viral entry into host cells is a particularly promising strategy since it halts infection at an early stage. Additionally, the long-term use of antiretroviral drugs has led to drug-resistant HIV strains, highlighting the need for new treatments, particularly those that block viral entry. HIV-1 uses envelope proteins gp120 and gp41 to attach to and enter host cells. Gp120 binds to the CD4 receptor on T-cells, then undergoes changes that allow it to interact with CCR5 or CXCR4 co-receptors. The interaction between the HIV-1 envelope glycoprotein gp120 and the primary host cell receptor CD4 is critical for viral entry and syncytium formation and therefore gp120 is a key target for developing entry inhibitors [79]. Various small molecules, peptides, antibodies, and recombinant proteins have shown potential in disrupting gp120 and co-receptor interactions. Additionally, the gp120 protein is rich in high-mannose oligosaccharides, which are crucial for infection [80]. Therefore, mannose-based molecules, glycodendrimers or glycodendrons, where explored as inhibitors of gp120 [81,82,83]. Kensinger and Wells demonstrated the potential of sulfated glycodendrimers to bind gp120, with lactose-based compounds showing notable activity [84]. Based on their research results newly designed mannose-based glycodendrimers (Man-DDs) and glycodendrons (Man-DNs) were synthesized. These compounds, with varied structures and multivalency, were created using efficient microwave-assisted click chemistry. Their interactions with the gp120 protein were analyzed using surface plasmon resonance and molecular modeling, showing potential as novel HIV entry inhibitors. Small mannose-based glycodendrons, particularly the divalent types, showed the strongest binding to the HIV-1 gp120 envelope protein. The most effective compounds featured either a methoxymethyl or azidomethyl group at their central position [85].

In addition to glycodendrimers, smaller glycoconjugates with antiviral activity have also been described. Inhibitors of N-glycosylation, such as 2-deoxy-d-glucose and 2-fluoro-2-deoxy-d-mannose, can prevent proper glycosylation of viral envelope proteins, resulting in non-fusogenic virus particles with reduced infectivity and impaired cell-to-cell transmission [86]. Furthermore, mannoside glycolipid conjugates consisting of a mannose headgroup, a hydrophilic linker, and a lipid chain, can exist as single molecules, dynamic micelles, and photopolymerized rigid polymers. Comparative studies revealed that dynamic micelles of trivalent conjugates exhibit superior inhibition of HIV-1 trans-infection compared to single molecules or rigid polymers, despite polymers not showing increased DC-SIGN binding affinity. The antiviral efficacy correlates strongly with the molecular assembly and structural flexibility of the conjugates, with dynamic micelles engaging both mannose residues and lipid chains for optimal interaction [87].

Targeted drug delivery systems with glycosylated structures were also investigated in order to enhance the efficacy and reduce their toxicity of antiviral agents, such as antiretroviral agents such as 3′-azido-3′-deoxythymidine (AZT) [88]. AZT 5′-monophosphate (AZTMP) was covalently conjugated to sugar-modified human serum albumin (HSA) to exploit lectin-mediated uptake pathways on T cells. Neoglycoproteins containing mannose, fucose, galactose, or glucose were synthesized and evaluated for antiviral activity. Only a high-mannose derivative (Man40HSA) exhibited intrinsic anti-HIV-1 activity, likely due to interference with the gp120-CD4 interaction. The Man22HSA-AZTMP conjugate showed over 30-fold greater activity than HSA-AZTMP and higher selectivity indices than free AZT or AZTMP [88].

CBAs also represent a promising class of anti-HIV compounds. They target the mannose-rich glycan structures on the HIV-1 envelope glycoprotein gp120. By binding to these glycans, CBAs block HIV-1 capture by DCs via the DC-SIGN, thereby preventing viral transmission to CD4+ T cells [89]. Examples of mannose-specific lectins that bind gp120 and prevent its interaction with CD4 are the griffithsin [90], the nematode-derived C-type lectin Mermaid [91] and the actinohivin, a protein isolated from the actinomycete *Longispora albida* [92].

### 3.2. Ebola Virus

Ebola virus (Ebo) is responsible for severe hemorrhagic fever with high fatality rates in humans, yet effective treatments remain unavailable. The fight against Ebola virus infection employs multiple strategies [93]. One approach includes the antiviral activity of CBAs. For example, a protein cyanovirin-N that shows potent inhibition of diverse HIV strains by targeting viral entry (high-mannose oligosaccharides on the HIV envelope glycoprotein gp120). Binding assays confirmed that cyanovirin-N interacts also with high affinity to the Ebola surface glycoprotein GP1,2 through a carbohydrate-dependent mechanism. Experimental studies have demonstrated that cyanovirin-N exhibits potent antiviral activity against the Zaire strain of Ebola virus (Ebo-Z), both in vitro and in vivo [94].

**Figure 8:**
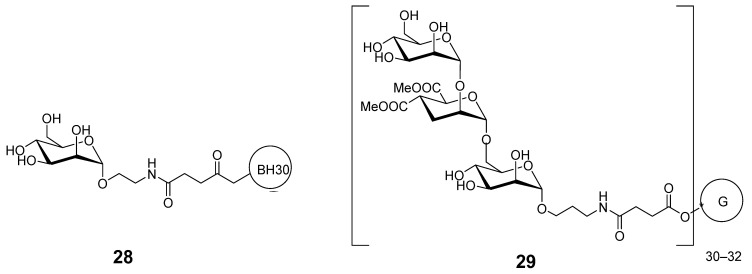
Multivalent inhibitors of Ebola virus infection.

Another strategy is prevention of pathogen entry by blocking cell-surface lectin receptors by multivalent carbohydrate-based compounds. This approach includes synthesizing compounds with sufficient size and multivalency to effectively mimic natural viral structures. Compound BH30sucMan **28** (Figure 8) based on BoltornH30 [95] was coupled with 32 mannose units linked through a succinyl spacer. In a model of Ebola virus infection that uses defective retroviral particles pseudotyped with Ebola virus envelope glycoprotein (EBOV-GP), BH30sucMan inhibited direct DC-SIGN-mediated cell entry [96].

Multivalent glycomimetics based on polyester dendrimers **29** (Figure 8) lead also to very potent inhibitors (in the nanomolar range) of cell infection by Ebola pseudotyped viral particles by blocking DC-SIGN receptor [97].

Furthermore, hexakis adducts of [59] fullerene provide an advantageous scaffold due to their stable globular shape and tunable size and valency. The water-soluble tridecafullerenes, termed “superballs”, decorated with 120 peripheral mannose moieties, exhibit potent antiviral activity (IC_50_ in subnanomolar range) in a model Ebola virus in the [98].

**Figure 9:**
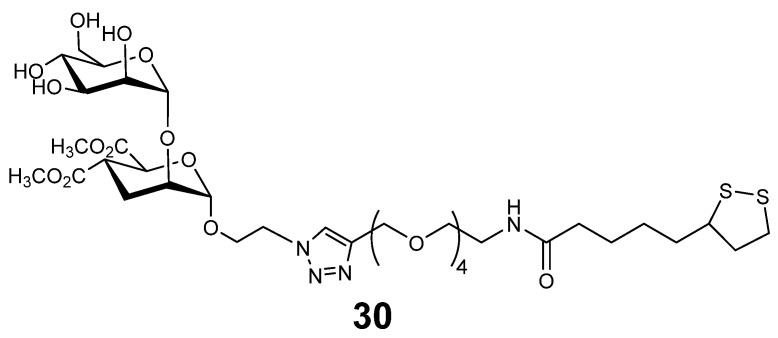
DC-SIGN ligand used for the preparation of mannosylated gold nanoparticles.

In addition to the aforementioned glycodendrimers and nanospheres “superballs, multivalent structures with antiviral activity can also be prepared in the form of gold nanoparticles. Synthetic pseudodimannose (psDiMan) ligand **30** (Figure 9) has been identified that selectively binds the DC-SIGN and was displayed in a multivalent fashion on gold nanoparticles of different diameters which bound DC-SIGN with sub-nanomolar dissociation constants. Gold-psDiMan nanoparticles inhibited DC-SIGN–mediated enhancement of Ebola pseudovirus entry with sub-nanomolar EC_50_ values [99].

### 3.3. Severe Acute Respiratory Syndrome Coronavirus 2 (SARS-CoV-2)

SARS-CoV-2 uses spike (S) protein is a homotrimeric transmembrane protein essential for viral entry into host cells, initiating entry into target cells. It is extensively glycosylated with N-linked oligomannose and complex sugars. ACE2 is the primary receptor for spike-mediated infection, but the virus can also interact with C-type lectin receptors such as DC-SIGN, which are expressed on antigen-presenting cells in the respiratory tract [100]. DC-SIGN binds to specific glycans present on the heavily glycosylated spike protein via multiple contact points. Although this interaction does not lead to direct infection of DC-SIGN–bearing cells, it enables these cells to capture the virus and transfer it to ACE2-expressing permissive cells, thereby enhancing viral dissemination. Blocking DC-SIGN with glycomimetic inhibitors can disrupt this transfer pathway, representing a potential antiviral strategy [101].

**Figure 10:**
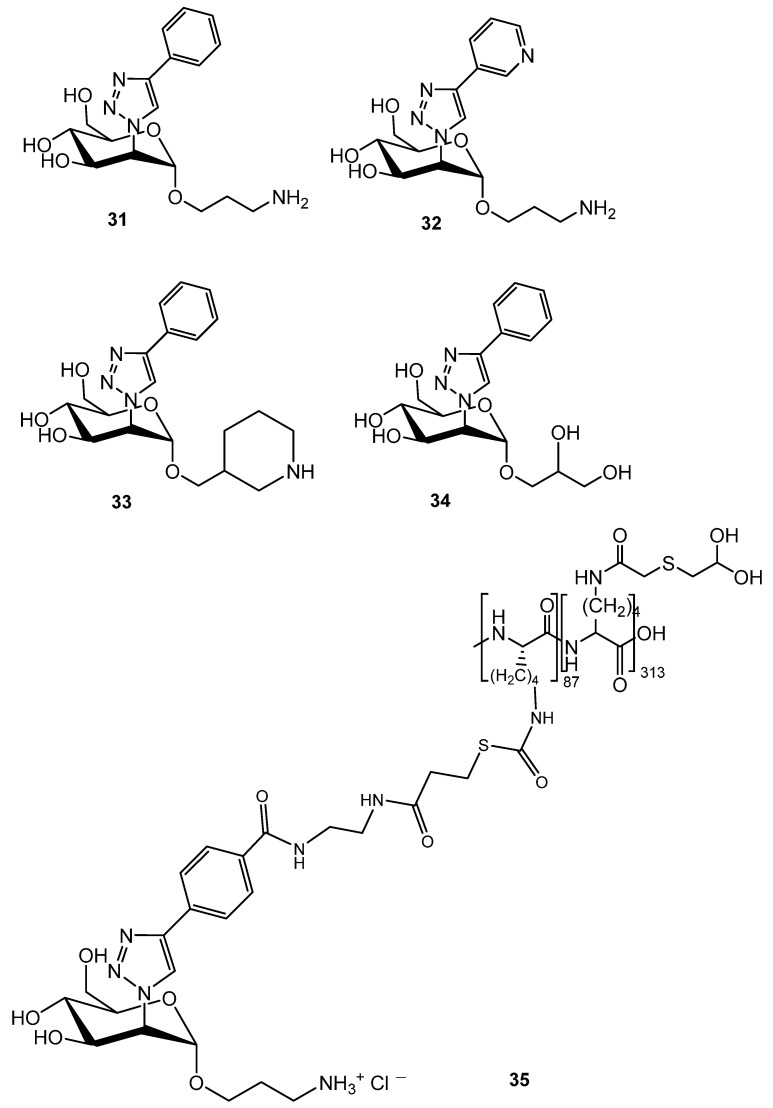
Lead triazole-based DC-SIGN antagonist.

Ernst et al. have identified triazole-based mannose analogues (including **31**–**34** show in Figure 10) as potent glycomimetic DC-SIGN antagonists. Compound **31** (Figure 10) was a lead glycomimetic ligand exhibiting over a 100-fold increase in binding affinity compared to methyl α-d-mannopyranoside. This ligand was further utilized to synthesize multivalent glycopolymer capable of inhibiting SARS-CoV-2 spike protein binding to DC-SIGN-expressing cells and blocking DC-SIGN-mediated trans-infection of ACE2-positive cells by spike protein-bearing viruses at nanomolar concentrations [102].

Gycopolymer **35** was found to inhibit the interaction of SARS-CoV-2 spike glycoprotein with DC-SIGN expressing cells with an IC_50_ of 4.4 nM, representing a 1.5 × 10^6^ improved binding affinity compared with the monovalent control MeMan.

**Figure 11:**
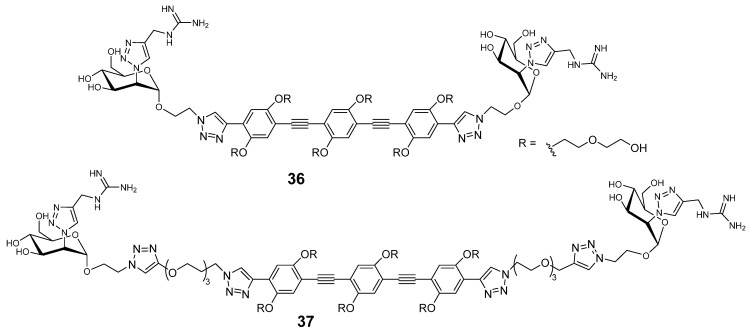
Divalent guanidine triazole mannoside selective for L-SIGN.

L-SIGN is closely related C-type lectin receptor to DC-SIGN. L-SIGN is found on airway epithelial endothelial cells and is not directly involved in immune regulation. Since severe COVID-19 is driven largely by immune overactivation, targeting L-SIGN, which is co-localized with ACE2-positive cells in the respiratory tract, may offer a safer route for anti-adhesion therapy. However, selective inhibition is complicated by the high similarity between the CRDs of DC-SIGN and L-SIGN [103]. Fieschi and Bernardi with coworkers have recently developed a mannose derivative carrying a methylene guanidine triazole at position 2, which binds L-SIGN and exhibits 50-fold selectivity over DC-SIGN. X-ray crystallography revealed that the guanidinium group provides steric and electrostatic complementarity specific to L-SIGN, a property traced to a single amino acid difference in the CRDs. Dimeric variants **36** and **37** (Figure 11) with short and long linkers demonstrated even greater selectivity and avidity, reaching low nanomolar affinities [103].

The glycan structures on S-glycoprotein present also promising targets for CBAs. The detailed mechanisms of CBA–ligand interactions remain underexplored, partly due to potential adverse effects associated with some CBAs. Recent in silico studies have examined interactions between SARS-CoV-2 S glycoprotein glycans and various lectins. These computational findings provide valuable insights that could guide the rational design of novel CBA-based antivirals aimed at neutralizing SARS-CoV-2 [104].

### 3.4. Human Papillomavirus and Herpes Simplex

Initial virus attachment of human papillomavirus (HPV) and herpes simplex virus (HSV) includes interaction with heparan sulfate proteoglycans on the host cell surface or extracellular matrix. Polycationic compounds such as polyethylenimine, poly-L-lysine, poly-L-arginine, poly-L-histidine, polyamidoamines, can inhibit viral adsorption by electrostatically. Two amphoteric linear polyamidoamines, designated ISA23 and AGMA1, have been reported to possess exceptional cytocompatibility and proven to be effective as inhibitor for HPV-16 and HSV-2 infection [105,106].

Furthermore, partially mannosylation of ISA23 and AGMA1 gave four biocompatible linear polyamidoamines carrying different amounts of mannosyl-triazolyl pendants. All mannosylated polyamidoamines inhibited HIV, and Man-AGMA6.5 and Man-AGMA14.5 also retained the parent polymer’s HPV-16 and HSV-2 inhibitory effects, demonstrating broad-spectrum dual-action antiviral activity [107].

Sulfated synthetic glycopolymers containing mannose showed also the potential as inhibitors of viral binding. Glycopolymer with 86 sulphated mannose units show the potential for broader activity against HPV16, and several other human viruses, such as HSV and Influenza A Virus [108].

## 4. Antiparasitic Activities

Glycosylphosphatidylinositols (GPIs) are complex glycolipids located on the surface of *Plasmodium* parasites and are believed to act as toxins during malaria infection. These molecules can trigger immune responses and stimulate the production of anti-GPI antibodies that help neutralize their harmful effects. As a result, vaccines targeting GPIs through glycoconjugate formulations may offer protection against malaria-related disease. To examine the impact of three specific structural features of *Plasmodium* GPIs, Lepenies and Varón Silva with co-workers synthesized six distinct GPI fragments from *Plasmodium falciparum*. These fragments were linked to the CRM197 carrier protein and assessed for their ability to provoke immune responses and provide protection in a cerebral malaria mouse model (C57BL/6JRj). The level of protection appears to be influenced by both antibody-mediated and cellular immune responses, which in turn are shaped by factors such as glycan orientation, mannose content, and the inclusion of phosphoethanolamine and inositol groups [109].

In *Entamoeba histolytica*, the antiretroviral lectin cyanovirin-N specifically binds α-1,2-linked mannose residues on surface N-glycans, causing aggregation and capping of key glycoproteins including the Gal/GalNAc adherence lectin and *O*-phosphodiester-linked glycoproteins. This capping inhibits phagocytosis, a crucial virulence mechanism. Mass spectrometry of lectin-enriched proteins identified known virulence factors and numerous previously uncharacterized, abundant glycoproteins, many of which are unique and promising vaccine candidates [110].

## 5. Antifungal Activities

Fungal infections pose a health challenge, compounded by limited therapeutic options and rising drug resistance. *Candida albicans* is a common commensal organism capable of causing severe infections, particularly in immunocompromised patients.

Poláková et al. evaluated antifungal activities of selected alkyl and (thio)dodecyl hexopyranosides based on d-glucose, d-galactose, *N*-acetyl d-glucosamine, and d-mannose. Treatment of *C. albicans* biofilms with (thio)alkyl glycosides led to a marked reduction in fungal cell proliferation, with activity dependent on glycoside structure and concentration. The relative potency of the tested derivatives against the azole-sensitive strain (GalOC_12_ > GlcOC_12_ ≈ ManOC_12_ > GlcNAcOC_12_) differed from that observed for the multiazole-resistant strain (GlcOC_12_ > ManOC_12_ ≈ GalOC_12_ > GlcNAcOC_12_) [111].

**Figure 12:**
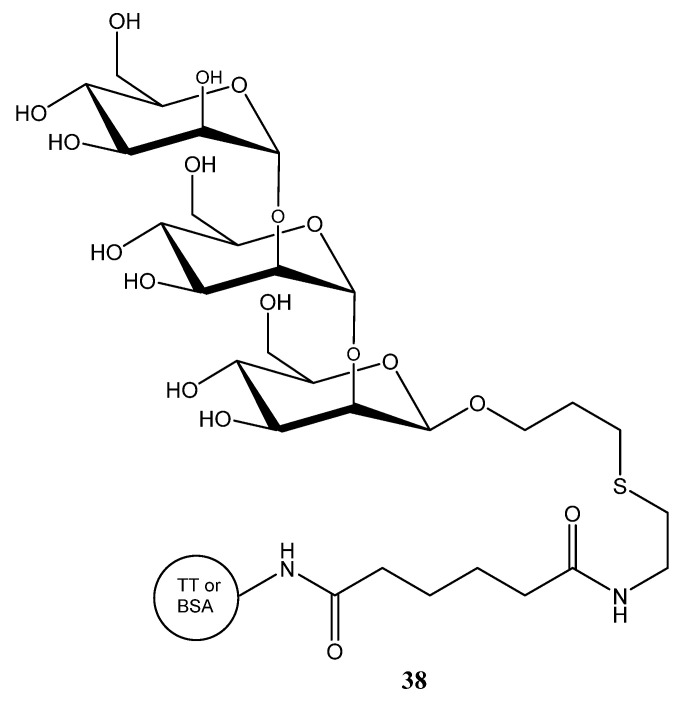
Trisaccharide conjugates used for the development of antifungal vaccines.

*C. albicans* strains can be classified into serotypes A and B, and further distinguished by antigenic factors 1, 4, 5, 6, and 13b. Most of these antigenic factors are composed of *β*-mannan chains, which differ in how they are attached to the α-mannan side chains of the cell wall. Consequently, the (1→2)-*β*-mannans located at the nonreducing ends of phosphomannan oligosaccharide side chains represent major antigenic determinants [112]. A minimal *β*-mannan disaccharide epitope from *C. albicans* that can elicit protective antibodies was identified and it was confirmed that a disaccharide or trisaccharide is sufficient for immune recognition and protection. Therefore, simple disaccharide or trisaccharide conjugates were linked to tetanus toxoid (TT) or bovine serum albumin (BSA) (Figure 12) [113].

Furthermore, peptide-based glycoconjugates incorporating known *Candida* T-cell epitopes were prepared [114], including a self-adjuvanting tricomponent vaccine linking *β*-mannan, T-cell peptide, and TT, which conferred protection in mice without added adjuvant [114]. Immunization in animal models demonstrated that these vaccines reduced fungal burden and improved survival. Notably, the combination of glycan and peptide (targeting both B and T cell responses) proved particularly effective [112].

Recently, Krylov and Nifantiev with coworkers have investigated two monoclonal antibodies, CM532 and FG70, which specifically recognize oligosaccharide fragments of fungal polysaccharides mannan and β-(1→3)-glucan, characteristic markers of fungal pathogens including *C. albicans*. CM532, developed by immunization with a pentamannoside conjugate, selectively binds a specific trisaccharide epitope in mannan. FG70, raised against a heptaglucan conjugate, interacts with a linear β-(1→3)-linked pentaglucoside fragment; branching in the epitope does not significantly affect this binding. These findings suggest that CM532 and FG70 have potential use for developing effective diagnostic tools for fungal infections, which are currently lacking [115].

## 6. Conclusions

Mannose represents a powerful and versatile building block in the development of next-generation anti-infective agents. Through its ability to engage both microbial adhesins and host lectin receptors, mannose-based conjugates have shown efficacy across a wide range of pathogens, including bacteria, viruses, fungi, and parasites. Advances in glycomimetic chemistry, nanotechnology, and vaccine design have expanded the scope of mannoconjugates from prophylactic anti-adhesion agents to targeted drug delivery systems and immunotherapeutics. Future efforts should focus on optimizing the structural features, multivalency, and pharmacokinetic properties of mannoconjugates, as well as validating their efficacy in clinically relevant models. Taken together, mannoconjugates hold substantial promise as innovative tools to address persistent challenges in infectious disease therapy and to contribute to the global fight against antimicrobial resistance.

## Abbreviations

The following abbreviations are used in this manuscript:

AZTMP	Azt 5′-Monophosphate
BH30	Boltornh30
BSA	Bovine Serum Albumin
CBA	Carbohydrate-Binding Agents
DC	Dendritic Cells
DC-SIGN	Dendritic Cell-Specific Intercellular Adhesion Molecule-3-Grabbing Non-Integrin
GPI	Glycosylphosphatidylinositol
HIV	Human Immunodeficiency Virus
HSA	Human Serum Albumin
IC_50_	half-maximal inhibitory concentration
Man	Mannose
αMeMan	methyl α-D-mannopyranoside
MIC	minimum inhibitory concentration
Mtb	Mycobacterium Tuberculosis
PEG	Polyethylene Glycol
SARS-CoV-2	Severe Acute Respiratory Syndrome Coronavirus 2
SLN	Solid Lipid Nanoparticles
Tb	Tuberculosis
TT	Tetanus Toxoid
UPEC	Uropathogenic *Escherichia Coli*
UTI	Urinary Tract Infection
